# Effects of dietary barley malt extract on growth performance, immune parameters, intestinal fermentation and gut microbiota in nursery piglets

**DOI:** 10.1007/s11250-026-05388-w

**Published:** 2026-10-10

**Authors:** Laya Kannan Silva Alves, Flávio de Aguiar Coelho, Rafaella Fernandes Carnevale, Matheus Saliba Monteiro, Janaina Cristina da Silva Maciel de Souza, Eduarda Buck Bernardes Guimarães, Cecília Archangelo Ferreira de Melo, Roberta Yukari Hoshino, Erick Marlon Pereira, Fernanda Mariane dos Santos, Nadia de Almeida Ciriaco Gomes, Ana Lígia Braga Mezzina, Caroline Veloso, José Alfonso Echavarria Martínez, Guilherme Marques Campora, Ana Clara Rodrigues de Oliveira, Allan Paul Schinckel, Monique Danielle Pairis-Garcia, Cesar Augusto Pospissil Garbossa

**Affiliations:** 1 Laboratory of Swine Research, Department of Nutrition and Animal Production, School of Veterinary Medicine and Animal Science, University of São Paulo, Pirassununga, 13635- 900 SP Brazil; 2 Animal Sciences Research and Education Center, Animal Sciences Faculty, College of Agriculture, Purdue University, West Lafayette, IN 47907-2053 USA; 3 Global Production Animal Welfare Laboratory, Department of Population Health and Pathobiology, College of Veterinary Medicine, North Carolina State University, Raleigh, NC 27607 USA

**Keywords:** Barley by-products, Diarrhea, Gut health, Short-chain fatty acids, Swine nutrition, Weaning

## Abstract

This study evaluated the effects of graded dietary barley malt extract (BME) supplementation on growth performance, fecal consistency, immune parameters, antioxidant status, short-chain fatty acid (SCFA) concentrations, and gut microbiota composition in nursery piglets. A total of 144 weaned piglets (24 days of age; 7.02 ± 1.08 kg) were allocated to four treatments containing 0, 0.33, 0.66, or 0.99% BME for 42 days. Performance variables were assessed at the pen level, and laboratory analyses were performed using one representative piglet per selected pen. No consistent dose–response effects were observed for body weight, average daily gain, feed intake, or diarrhea incidence throughout the nursery period. A linear increase in feed conversion ratio was detected during the pre-starter II phase (*P* = 0.023), but no cumulative performance effects were observed over the overall nursery period. Antioxidant enzyme activities, leukocyte populations, and fecal SCFA concentrations were unaffected by BME inclusion (*P* > 0.05). Microbial alpha diversity indices were unchanged; however, specific microbial taxa, including Veillonellaceae, *Mitsuokella*, and *Mogibacterium*, differed among inclusion levels. Overall, dietary BME up to 0.99% did not improve growth performance, immune parameters, antioxidant status, or intestinal fermentation in nursery piglets, although modest shifts in microbial composition were observed.

**Supplementary Information:**

The online version contains supplementary material available at https://doi.org/10.1007/s11250-026-05388-w.

## Highlights

• Graded inclusion of barley malt extract was evaluated in nursery piglets.

• BME up to 0.99% did not improve growth performance or feed efficiency.

• Immune parameters, antioxidant status and SCFA were unaffected by BME.

• Specific microbial taxa shifted without functional performance effects.

• Barley malt extract appears nutritionally neutral under nursery conditions.

**Supplementary Information:**

The online version contains supplementary material available at https://doi.org/10.1007/s11250-026-05388-w.

## Introduction

Barley (*Hordeum vulgare* L.) is one of the most cultivated cereals worldwide and is processed primarily for malt production (FAO 2024). During malting, biochemical modifications increase the availability of soluble nutrients within the grain, resulting in barley malt extract (BME) composed predominantly of simple sugars and amino acids (Celus et al. 2006; Gupta et al. 2010; Rani and Bhardwaj 2021). Barley malt extract is widely used in the food and beverage industry, particularly in beer production and fermented products (Martínez et al. 2018). More recently, interest has expanded toward its potential application in animal nutrition due to its content of fermentable carbohydrates, soluble proteins, and bioactive compounds, including phenolic compounds and β-glucans (Newman and Newman 2008; Šimić et al. 2019). These components have been associated with modulation of gut microbiota and immune responses, and previous studies have reported improvements in growth performance and meat quality in poultry (Sedghi et al. 2018).

Research evaluating barley malt-derived ingredients in swine nutrition remains limited and inconsistent. While malted barley grain has shown potential benefits in growing-finishing pigs (Nasir et al. 2015), other studies have reported impaired feed efficiency when brewery by-products or powdered malt were included in diets (Beyihayo et al., 2022). Because available evidence is limited to specific production stages, primarily finishing pigs, the potential benefits or limitations of BME supplementation across other production phases remain unclear. To the authors’ knowledge, no studies have evaluated dietary BME supplementation in weaned piglets.

Weaned piglets represent a nutritionally vulnerable population, as weaning induces morphological and functional alterations in the gastrointestinal tract, reduces voluntary feed intake, and temporarily compromises nutrient absorption and immune competence (Tang et al. 2022). This post-weaning period is commonly characterized by impaired growth performance and increased susceptibility to enteric disturbances (Han et al. 2024). These challenges may be further exacerbated under commercial tropical production conditions, where environmental and sanitary stressors can intensify physiological strain (Fan et al. 2025). Thus, nutritional strategies capable of supporting intestinal function and promoting adaptation during the post-weaning transition remain of interest.

Because BME contains readily digestible carbohydrates and soluble proteins derived from the endosperm (Bhatty 1999; Delcour and Hoseney 2010), its inclusion in nursery diets may support nutrient availability during the post-weaning period (Zheng et al. 2021). Therefore, this study aimed to evaluate the effects of graded dietary inclusion levels (0, 0.33, 0.66 and 0.99%) of BME on growth performance, immune parameters, antioxidant status, intestinal fermentation, and gut microbiota in nursery piglets.

## Materials and methods

The experimental protocol was approved by the Animal Use Ethics Committee of the School of Veterinary Medicine and Animal Science of the University of São Paulo (protocol no. 892250620). The study was conducted at the Laboratory of Swine Research, University of São Paulo, Pirassununga, São Paulo, Brazil (21°59′46″ S, 47°25′36″ W).

### Animals, housing, and experimental design

A total of 144 *Danbred Brasil* (DB90 x LQ1250) weaned piglets (72 barrows and 72 gilts) with an average initial body weight of 7.02 ± 1.08 kg were used in this study. Piglets were weaned at 24 days of age and housed in same-sex pens, with three barrows or three gilts per pen. Each pen provided a usable floor area of 1.08 m², corresponding to a floor space allowance of 0.36 m² per piglet and a stocking density of 2.78 piglets/m², and was equipped with partially slatted floors, a semi-automatic feeder, and a nipple drinker. Feed and water were provided *ad libitum* throughout the 42-day experimental period, which was divided into three feeding phases: pre-starter I (days 0–7), pre-starter II (days 8–28), and starter (days 29–42).

Room temperature and relative humidity were monitored daily and recorded throughout the study. Environmental conditions were manually adjusted using curtains and heat lamps to maintain thermal comfort according to recommendations for nursery pigs (Brown-Brandl et al. 2023). Detailed environmental records are presented as supplementary material (Figure S1).

The experiment followed a randomized complete block design, with sex and initial body weight used as blocking factors to minimize baseline variability among experimental units. Piglets were allocated to one of four dietary treatments, with 12 replicate pens per treatment (three piglets per pen). The pen was considered the experimental unit for growth performance and diarrhea incidence analyses. The number of replicate pens was determined based on facility capacity and availability of experimental units within the nursery barn (48 pens total), while maintaining balanced treatment allocation across blocks.

Four dietary treatments were evaluated:

- CON: control diet, without the inclusion of BME.
- BME0.33%: inclusion of 0.33% BME in the diet.
- BME0.66%: inclusion of 0.66% BME in the diet.
- BME0.99%: inclusion of 0.99% BME in the diet.

Diets were formulated to meet or exceed the nutritional requirements of high-genetic-potential nursery piglets according to Rostagno et al. (2017). Ingredient and calculated nutrient compositions for each feeding phase are presented in Table 1. Barley malt extract was included at the expense of kaolin to maintain isoenergetic and isonitrogenous formulations. Inclusion levels (0.33–0.99%) were selected to provide graded increases in soluble nutrients without exceeding 1%, a level previously associated with functional effects in monogastric species.

**Table 1:** Ingredients and calculated nutrient composition of experimental diets fed to nursery piglets supplemented with graded levels of barley malt extract (BME) across feeding phases

Ingredient composition (% as-fed basis)	Pre-Starter I		Pre-Starter II	Starter
Corn	24.000		39.000	61.000
Soybean meal, 45%	15.000		20.000	27.000
Micronized whole soybean	9.000		1.750	7.000
Soy protein concentrate	6.000		6.250	-
Starch	23.449		15.821	0.757
Whey	12.097		6.250	-
Dairy product, 38% lactose	6.000		6.250	-
DL-methionine	0.227		0.187	0.101
L-tryptophan	0.052		0.040	0.008
L-threonine	0.185		0.158	0.091
L-lysine	0.500		0.434	0.312
L-valine	0.114		0.071	-
Phytase	0.010		0.010	0.010
Premix Vit/Min^1^	1.000		1.000	1.000
Salt	0.200		0.250	0.400
Limestone	-		-	0.579
Dicalcium phosphate	0.542		0.704	0.668
Calcium sulfate	0.550		0.750	-
Antioxidant	0.025		0.025	0.025
Flavoring	0.050		0.050	0.050
Kaolin / BME²	1.000		1.000	1.000
**Calculated composition**				
Metabolizable energy (kcal/kg)	3694		3607	3259
Crude protein (%)	21.48		19.08	20.44
Total lysine (%)	1.51		1.44	1.32
Digestible lysine (%)	1.45		1.39	1.17
Total methionine + cysteine (%)	0.90		0.84	0.55
Digestible methionine + cysteine (%)	0.81		0.75	0.49
Ca (%)	0.73		0.63	0.60
P (%)	0.53		0.48	0.37
Lactose (%)	15.45		9.98	-
**Dry barley malt extract (BME) composition (% of dry matter**,** unless stated)**				
*Item*			*DME*	
DM (% as-fed)			86.8	
Organic matter			98.2	
Neutral detergent fiber			2.12	
Ether extract			0.45	
Crude protein			8.17	
Reducing sugars			71.0	
Fructose			0.84	
Glucose			3.54	
Sucrose			0.94	
Maltose			31.7	

^1^Composition per kg of premix: Cu (12 mg); Fe (80 mg); I (1 mg); Mn (40 mg); Se (0.36 mg); Zn (110 mg); vit. A (6875 I.U.); vit. D3 (1505. I.U.); vit. E (40 mg); vit. K3 (3.07 mg); vit. B1 (1 mg); vit. B2 (3.13 mg); vit. B6 (2 mg); vit. B12 (0.02 mg); niacin (30 mg); folic acid (0.3 mg); pantothenic acid (15 mg); biotin (0.1 mg); choline (200.97 mg).² Kaolin was progressively replaced by barley malt extract (BME) according to treatment level (0, 0.33, 0.66, or 0.99%)

### Growth performance

Piglets were individually weighed at the beginning of the trial (day 0) and on days 7, 28, and 42, corresponding to the end of each feeding phase. Body weight (BW) was recorded individually, and performance variables were calculated on a pen basis, which was considered the experimental unit. Average daily gain (ADG) was calculated as the difference between final and initial body weight within each period divided by the number of experimental days. Average daily feed intake (ADFI) was calculated as feed offered minus feed refusals, divided by the number of experimental days. Feed conversion ratio (FCR) was calculated as the ratio between ADFI and ADG.

### Diarrhea incidence

Fecal consistency was assessed according to Pedersen and Toft (2011) by visual inspection of feces in each pen. Evaluations were performed once daily at 08:00 h by the same observer (JCSMS) throughout the experimental period. Fecal scores ranged from 1 to 4: 1 = firm and shaped, 2 = soft and shaped, 3 = soft and unshaped, and 4 = liquid. Scores 1 and 2 were considered normal, whereas scores 3 and 4 were classified as diarrhea. Diarrhea incidence was calculated per pen as the percentage of daily observations classified as diarrhea (scores 3–4) within each feeding phase and cumulatively for the entire experimental period (days 0–42).

### Animal selection and sample collection

For immunological, antioxidant, short-chain fatty acid (SCFA), and microbiota analyses, eight pens per treatment were randomly selected from the total experimental units. Within each selected pen, one piglet whose body weight was closest to the pen average was chosen to provide a representative sample of the experimental unit and reduce within-pen variability.

Blood samples were collected on days 7, 28, and 42 for immunological analysis, whereas antioxidant status was evaluated only on day 28. Fecal samples were collected by rectal stimulation on day 28 for SCFA quantification and microbiota analysis. To minimize circadian variation, all biological samples were collected at similar times of day.

### Immunological parameters

Blood samples were collected via jugular venipuncture using sterile needles and EDTA-coated BD Vacutainer^®^ tubes. Approximately 10 mL of blood was obtained per piglet. Total and differential leukocyte counts were determined using an automatic hematology analyzer (BC2800Vet, Mindray, China). Differential leukocyte counts were expressed as absolute cell counts (cells/mm³). Blood smears were stained using the May-Grünwald-Giemsa method when morphological confirmation was required. Serum total protein concentration was determined by refractometry (Quimis, Brazil).

### Antioxidant status

Plasma obtained by centrifugation was stored at − 20 °C until analysis. Superoxide dismutase (SOD), glutathione S-transferase (GST), reduced glutathione peroxidase (GSH-Px), and lipid peroxidation (LPO) were analyzed according to the methodologies described by Jiang et al. (1991) and Grando et al. (2023).

### Short-chain fatty acid concentrations

Approximately 2 g of fresh fecal material was mixed with 4 mL of 17% formic acid solution, homogenized and centrifuged at 2,500 rpm. SCFA concentrations were determined using external calibration curves prepared with chromatographic standards (Chem Service, West Chester, PA, USA), as described by Ferreira et al. (2016).

### Microbiota analysis

Fresh fecal samples were collected by rectal stimulation, placed in sterile tubes, and immediately stored under appropriate conditions until DNA extraction. Genomic DNA was extracted using the ZR Fecal DNA Miniprep Kit (Zymo Research, Irvine, CA, USA), following the manufacturer’s instructions and the method described by Holman and Chénier (2013).

The V4 hypervariable region of the 16 S rRNA gene was amplified using primers 515-F and 806-R, targeting an approximately 250-bp fragment. PCR amplification consisted of an initial denaturation at 94 °C for 3 min, followed by 28 cycles of denaturation at 94 °C for 30 s, annealing at 53 °C for 40 s, and extension at 72 °C for 1 min, with a final extension at 72 °C for 5 min.

Amplicon size and specificity were verified by 1% agarose gel electrophoresis. Samples were pooled in equimolar concentrations and prepared for sequencing using the Illumina TruSeq DNA Library Preparation Kit (Illumina, San Diego, CA, USA). Sequencing was performed on an Illumina MiSeq platform (2 × 250-bp paired end reads) according to the manufacturer’s instructions.

Raw sequences were processed using the QIIME software package (version 1.8.0) (Caporaso et al. 2010). Sequences were clustered into operational taxonomic units (OTUs), and taxonomic classification was assigned by comparison against the SILVA ribosomal RNA gene database (release 128; Yilmaz et al. 2014).

### Statistical analysis

All statistical analyses were performed in RStudio software (version 4.2.1). Growth performance, diarrhea incidence, oxidative stress markers, and SCFA concentrations were analyzed using linear mixed-effects models. Dietary BME inclusion level was modeled as a continuous predictor to test linear and quadratic dose–response effects. Block, defined by sex and initial body weight, was included as a random effect in all models. For growth performance and diarrhea incidence, the pen was considered the experimental unit.

Hematological parameters were analyzed using mixed-effects models that included dietary BME inclusion level (linear and quadratic effects), experimental period (days 7, 28, and 42), and their interaction. Because no significant treatment × period interactions were detected, the interaction terms were removed from the final models. Absolute leukocyte counts (cells/mm^3^) were used in the analyses to improve biological interpretability.

Microbiota alpha diversity indices were analyzed using the Kruskal–Wallis test when normality assumptions were not met. When significant, pairwise comparisons were performed using Dunn’s test with adjustment for multiple comparisons. Relative abundance of selected taxa was analyzed using analysis of variance (ANOVA), considering treatment as a categorical fixed effect, followed by Tukey’s post hoc test.

Model assumptions were evaluated by visual inspection of residuals and by Shapiro–Wilk and Bartlett’s tests to assess normality and homoscedasticity, respectively. Variables that did not meet the model assumptions were transformed prior to analysis. Data are presented as estimated marginal means (EMMs), and the largest standard error of the mean (SEM) is reported. Statistical significance was declared at *P* < 0.05, whereas tendencies were considered at 0.05 ≤ *P* < 0.10.

## Results

### Growth performance

Growth performance results are presented in Table 2. During the pre-starter I phase (days 0–7), no linear or quadratic effects were detected for body weight, ADG, ADFI, or FCR (*P* > 0.05). A tendency for a quadratic effect was detected for ADG (*P* = 0.051), whereas tendencies for linear and quadratic effects were observed for ADFI (*P* = 0.070 and *P* = 0.088, respectively).

In the pre-starter II phase (days 8–28), FCR increased linearly with increasing dietary BME inclusion (*P* = 0.023). A tendency for a linear effect was observed for ADFI (*P* = 0.083), whereas body weight and ADG were unaffected (*P* > 0.05). During the starter phase (days 29–42), no linear or quadratic effects were observed for any performance variable (*P* > 0.05). Considering the overall experimental period (days 0–42), BME inclusion did not affect ADG, ADFI, or FCR (*P* > 0.05).

**Table 2:** Growth performance of nursery piglets supplemented with increasing dietary levels of barley malt extract (BME) during the nursery phase

Variable	Level of BME inclusion, %				SEM	*P*-value		
	0	0.33	0.66	0.99		Linear	Quadratic	
**Pre-Starter I (0–7 D)**								
Initial weight, kg	7.01	7.03	7.04	7.02	0.315	0.457		0.216
Weight at day 7, kg	7.99	7.98	8.18	8.02	0.373	0.563		0.134
ADG, kg	0.142	0.137	0.166	0.141	0.010	0.468		0.051
ADFI, kg	0.184	0.195	0.203	0.188	0.007	0.070		0.088
FCR	1.353	1.603	1.301	1.391	0.077	0.410		0.243
**Pre-starter II (8-28D)**								
Weight at day 28, kg	15.42	15.69	15.65	15.70	0.637	0.801		0.478
ADG, kg	0.359	0.367	0.359	0.373	0.013	0.903		0.836
ADFI, kg	0.570	0.608	0.603	0.611	0.027	0.083		0.771
FCR	1.643	1.655	1.681	1.643	0.031	0.023		0.806
**Starter (29-42D)**								
Weight at day 42, kg	21.79	21.57	22.25	21.95	1.393	0.525		0.807
ADG, kg	0.455	0.420	0.472	0.445	0.077	0.660		0.856
ADFI, kg	0.812	0.874	0.841	0.808	0.068	0.830		0.893
FCR	1.994	2.037	1.802	1.809	0.126	0.127		0.184
**Total Experimental Period (0-42D)**								
ADG, kg	0.352	0.346	0.362	0.356	0.022	0.727		0.639
ADFI, kg	0.585	0.628	0.612	0.605	0.031	0.380		0.745
FCR	1.715	1.769	1.690	1.691	0.040	0.466		0.555

Piglets were evaluated during pre-starter I (days 0–7), pre-starter II (days 8–28), starter (days 29–42), and across the total experimental period (days 0–42). Dietary BME inclusion level was modeled as a continuous predictor to test linear and quadratic dose-response effects. SEM: standard error of the mean. ADG: average daily gain; ADFI: average daily feed intake; FCR: feed conversion ratio

### Diarrhea incidence

**Fig. 1:**
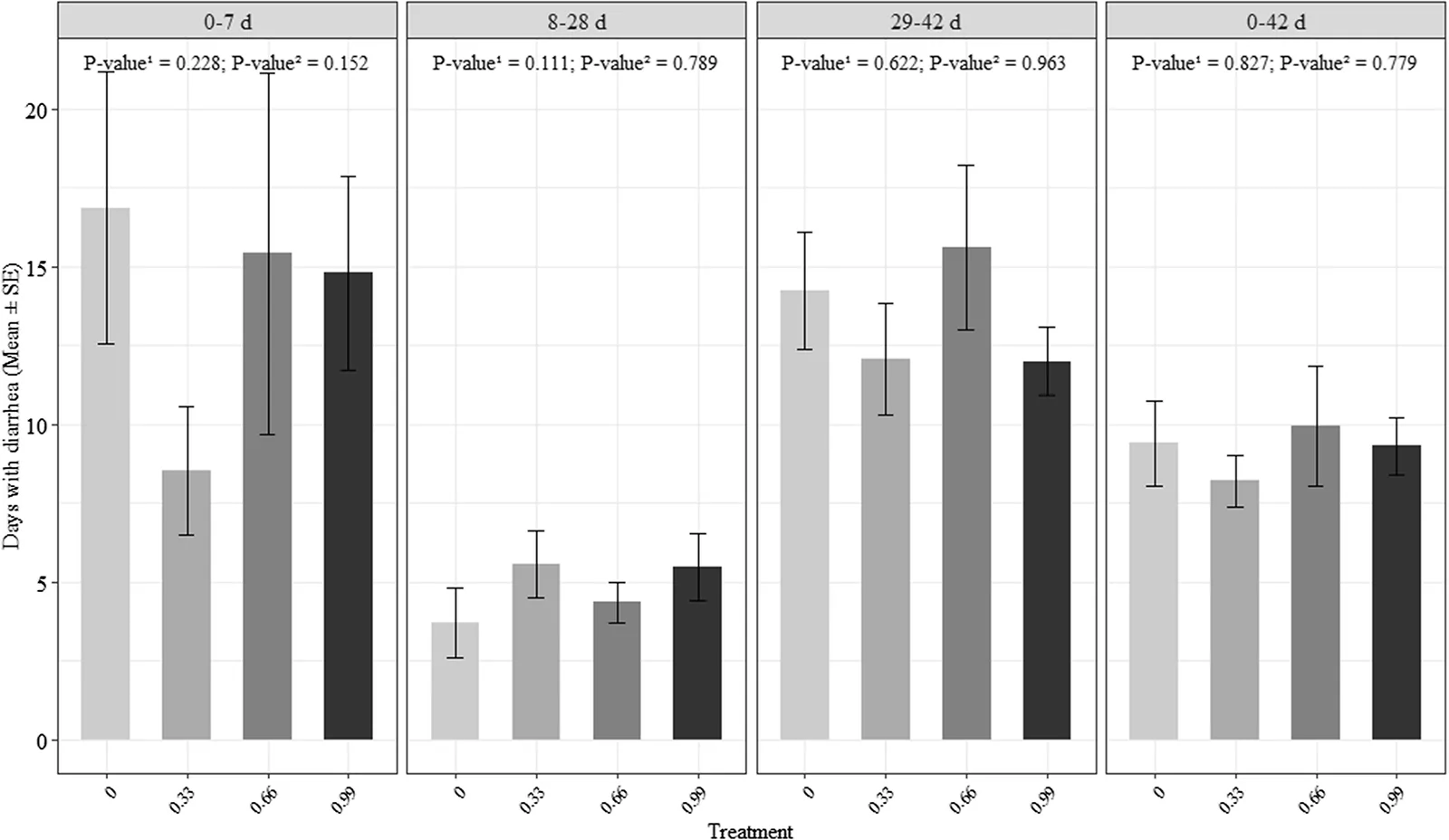
Diarrhea incidence (%) in nursery piglets supplemented with increasing levels of barley malt extract (BME) during the nursery phase. Diarrhea incidence was calculated as the percentage of daily fecal observations classified as diarrhea (scores 3–4) according to Pedersen and Toft (2011), within each feeding phase and cumulatively (days 0–42). Dietary BME inclusion level was modeled as a continuous predictor to test linear and quadratic effects. P-value¹ and P-value² correspond to the linear and quadratic effects, respectively

Diarrhea incidence results are presented in Fig. 1. No linear or quadratic effects of BME inclusion were observed during any feeding phase or over the entire experimental period (days 0–42, *P* > 0.05).

### Immunological parameters

Immunological results are presented in Table 3. No linear or quadratic effects of BME inclusion were observed for mean platelet volume, neutrophils, monocytes, eosinophils, or basophils (*P* > 0.05). For lymphocytes, no linear effect was detected (*P* = 0.731); however, a tendency for a quadratic effect was observed (*P* = 0.075). An experimental period effect was observed for neutrophils (*P* < 0.001), lymphocytes (*P* = 0.017), and basophils (*P* = 0.004), reflecting physiological variations over time.

**Table 3:** Hematological parameters of nursery piglets supplemented with increasing dietary levels of barley malt extract (BME) during the nursery phase

Variables	Period	Level of BME inclusion, %				Mean	SEM	*P*-value			
		0	0.33	0.66	0.99			Period	Linear	Quadratic	
Mean platelet volume (elements/mm^3^)	7d	383.00	340.00	481.00	472.00	419.34	544.52	0.714	0.340	0.186	
	28d	439.00	431.00	287.20	428.75	396.529					
	42d	352.13	523.52	451.63	413.28	439.139					
	Mean	391.37	431.70	406.89	438.05						
Neutrophils (cells x 10^4^/mm^3^)	7d	10.992	6.442	8.703	9.479	8.903	1.652	< 0.0001	0.649	0.176	
	28d	6.034	4.060	4.712	6.659	5.366					
	42d	9.278	9.143	8.134	8.353	8.763					
	Mean	8.767	6.548	7.183	8.163						
Lymphocytes (cells x 10^4^/mm^3^)	7d	9.664	8.518	10.111	10.703	9.749	1.199	0.017	0.731	0.075	
	28d	9.756	12.307	13.958	9.850	11.965					
	42d	9.775	9.800	11.290	10.526	10.348					
	Mean	9.733	10.208	11.786	11.021						
Monocytes (cells x 10^4^/mm^3^)	7d	0.732	0.562	0.689	0.701	0.671	0.205	0.160	0.802	0.908	
	28d	0.871	0.649	0.787	0.486	0.698					
	42d	0.554	0.743	0.979	0.786	0.765					
	Mean	0.719	0.651	0.818	0.657						
Eosinophils (cells x 10^4^/mm^3^)	7d	0.239	0.166	0.208	0.195	0.202	0.045	0.469	0.289	0.514	
	28d	0.199	0.201	0.231	0.270	0.225					
	42d	0.176	0.147	0.236	0.192	0.188					
	Mean	0.205	0.171	0.225	0.219						
Basophils (cells x 10^4^/mm^3^)	7d	1.365	1.141	1.412	1.619	1.384	1.26	0.004	0.631	0.808	
	28d	0.583	0.524	1.089	0.952	0.786					
	42d	1.179	1.500	1.178	1.049	1.226					
	Mean	1.042	1.055	1.226	1.206						

Blood samples were collected on days 7, 28, and 42. Dietary BME inclusion level was modeled to test linear and quadratic dose–response effects. Experimental period corresponds to sampling day. Data are expressed as estimated marginal means (EMMs). SEM = largest standard error of the mean

### Antioxidant status

Antioxidant status results are presented in Table 4. No linear or quadratic effects of BME inclusion were observed for plasma glutathione peroxidase, lipid peroxidation, glutathione S-transferase, or superoxide dismutase activity (*P* > 0.05). Total protein concentration was not affected; however, a trend for a quadratic effect was observed (*P* = 0.091).

**Table 4:** Plasma antioxidant status indicators of nursery piglets supplemented with increasing dietary levels of barley malt extract (BME)

Variables	Level of BME inclusion, %				SEM	*P*-value	
	0	0.33	0.66	0.99		Linear	Quadratic
GSH-Px, µg/g	58.77	58.77	51.81	52.93	4.75	0.291	0.887
LPO, nmol/min/mg protein	133.93	169.35	166.93	189.86	48.87	0.276	0.997
GST, mmol/min/mg protein	0.18	0.07	0.09	0.03	0.058	0.131	0.304
SOD, U/mg protein	332.05	336.82	345.48	337.35	15.18	0.665	0.105
Total protein, mg/mL	2.36	2.47	2.46	2.39	0.051	0.770	0.091

Samples were collected on day 28 of the nursery phase. Dietary BME inclusion level was modeled as a continuous predictor to test linear and quadratic effects. Data are expressed as estimated marginal means (EMMs). SEM: standard error of the mean; GSH-Px: glutathione peroxidase; LPO: lipid peroxidation; GST: glutathione S-transferase; SOD: superoxide dismutase

### Short-chain fatty acid concentrations

Short-chain fatty acid concentrations are presented in Table 5. No linear or quadratic effects of BME inclusion were observed for acetate, propionate, isobutyrate, butyrate, isovalerate, or valerate concentrations (*P* > 0.05).

**Table 5:** Fecal short-chain fatty acid (SCFA) concentrations of nursery piglets supplemented with increasing dietary levels of barley malt extract (BME)

Variables (mM/L)	Level of BME inclusion, %				SEM	*P*-value	
	0	0.33	0.66	0.99		Linear	Quadratic
Acetate	23.00	25.47	24.18	25.23	1.483	0.888	0.411
Propionate	9.40	9.21	10.29	9.35	0.477	0.576	0.200
Isobutyrate	0.71	0.68	0.69	0.74	0.049	0.306	0.249
Butyrate	5.76	6.41	5.29	5.32	0.533	0.310	0.488
Isovalerate	1.04	1.00	1.00	1.05	0.087	0.921	0.686
Valerate	1.77	1.56	1.70	1.44	0.165	0.480	0.920

Fecal samples were collected on day 28. Dietary BME inclusion level was modeled as a continuous predictor to test linear and quadratic effects. Data are expressed as estimated marginal means (EMMs). SEM: largest standard error of the mean

### Microbiota analysis

**Fig. 2:**
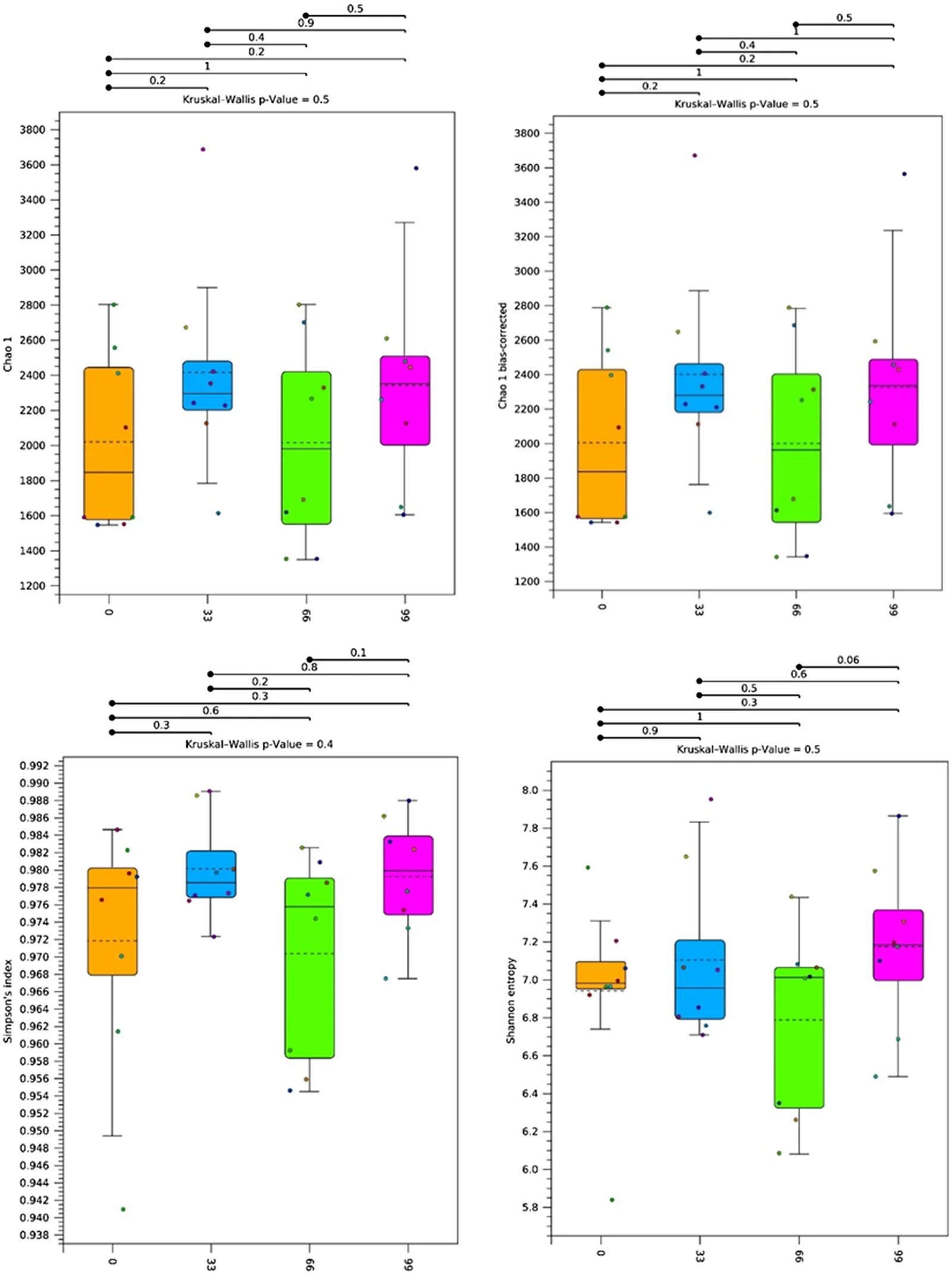
Alpha diversity indices (Chao1, corrected Chao1, Simpson, and Shannon) of fecal microbiota in nursery piglets supplemented with increasing dietary levels of barley malt extract (BME). Fecal samples were collected on day 28 of the nursery phase. Differences among treatments were evaluated using the Kruskal–Wallis test at the 5% significance level. Box plots display the median (line), interquartile range (box), whiskers extending to 1.5 × IQR, and outliers (“+”). IQR: interquartile range. Treatment labels 0, 33, 66, and 99 correspond to 0, 0.33, 0.66, and 0.99% BME, respectively

**Fig. 3:**
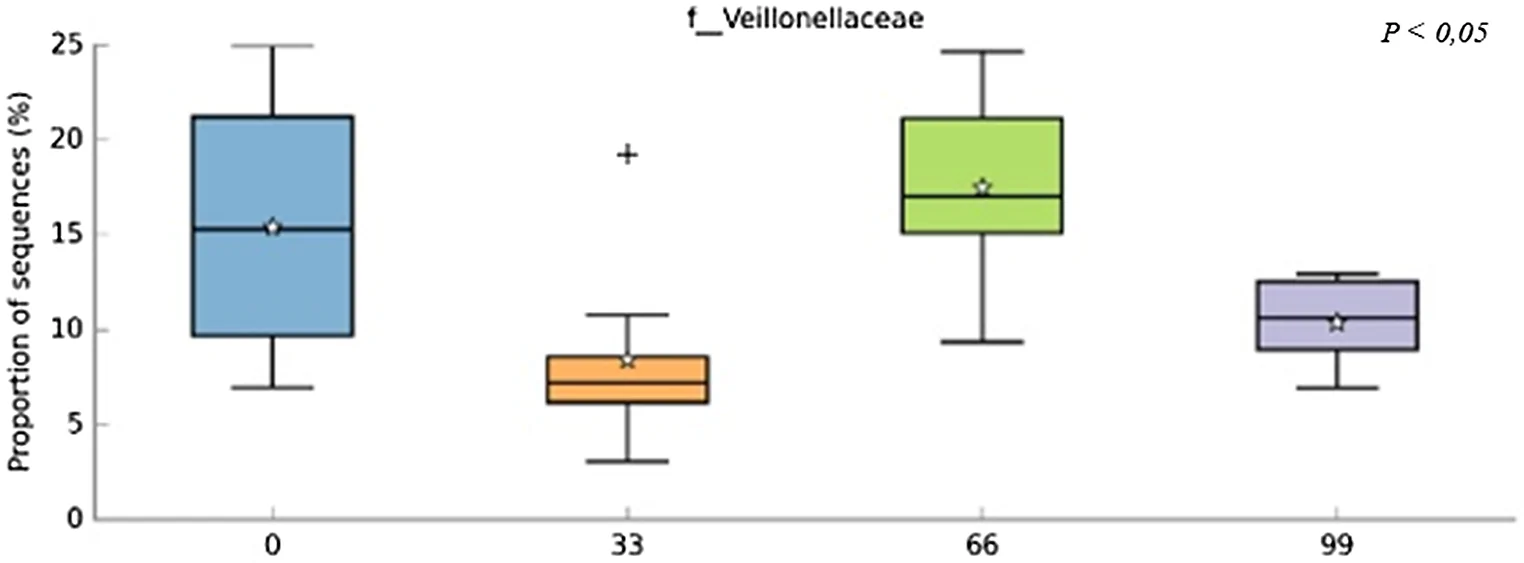
Relative abundance of the family Veillonellaceae in fecal samples of nursery piglets supplemented with increasing dietary levels of barley malt extract (BME). Fecal samples were collected on day 28 of the nursery phase. Means were compared using ANOVA followed by Tukey’s test at the 5% significance level. Box plots display the median (line), mean (star), interquartile range (box), whiskers extending to 1.5 × IQR, and outliers (“+”). IQR: interquartile range. Treatment labels 0, 33, 66, and 99 correspond to 0, 0.33, 0.66, and 0.99% BME, respectively

**Fig. 4:**
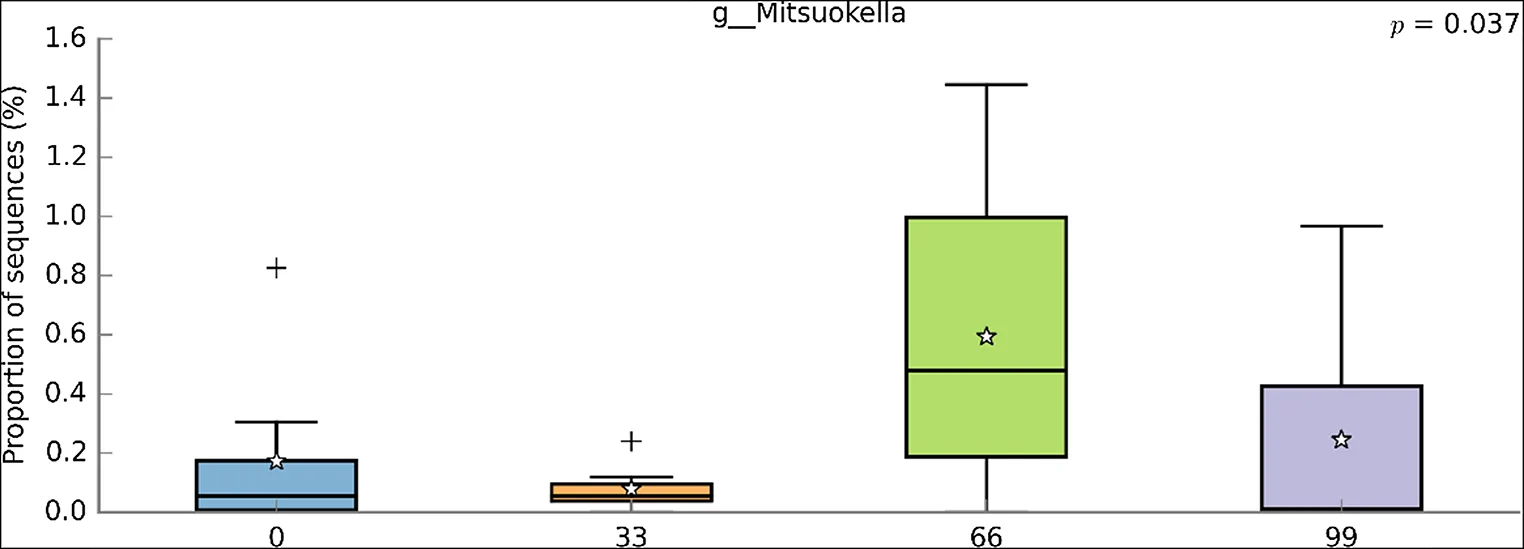
Relative abundance of the genus Mitsuokella in fecal samples of nursery piglets supplemented with increasing dietary levels of barley malt extract (BME). Fecal samples were collected on day 28 of the nursery phase. Means were compared using ANOVA followed by Tukey’s test at the 5% significance level. Box plots display the median (line), mean (star), interquartile range (box), whiskers extending to 1.5 × IQR, and outliers (“+”).IQR: interquartile range. Treatment labels 0, 33, 66, and 99 correspond to 0, 0.33, 0.66, and 0.99% BME, respectively

**Fig. 5:**
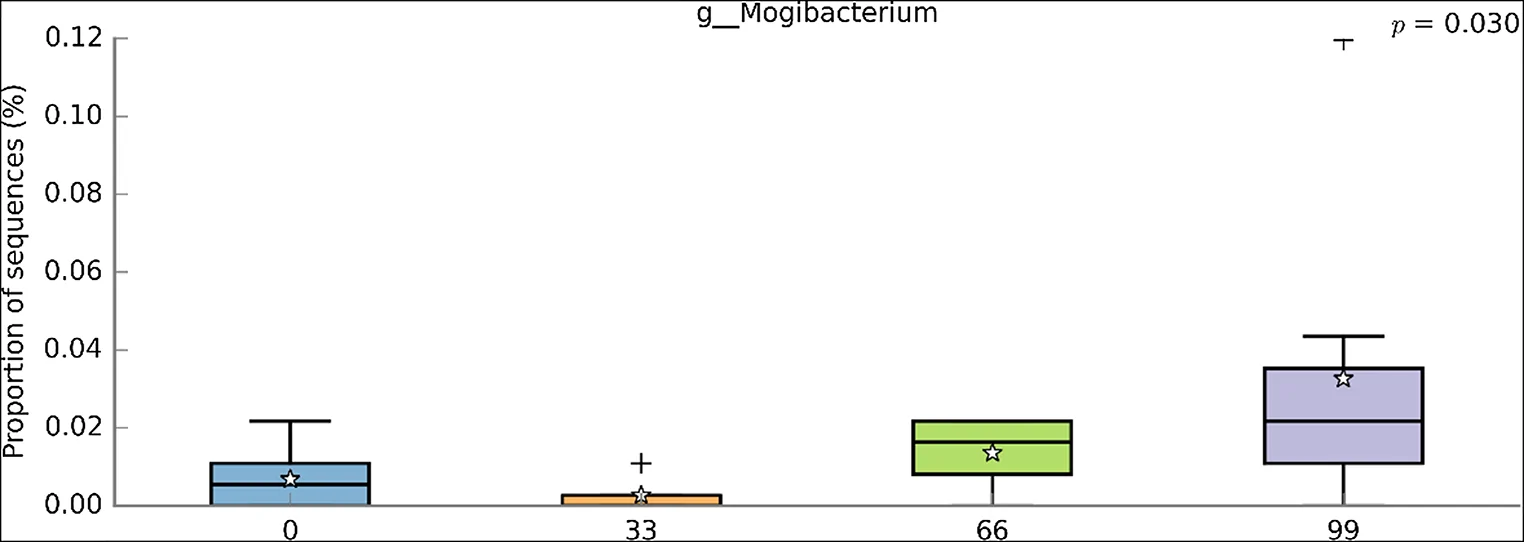
Relative abundance of the genus *Mogibacterium* in fecal samples of nursery piglets supplemented with increasing dietary levels of barley malt extract (BME). Fecal samples were collected on day 28 of the nursery phase. Means were compared using ANOVA followed by Tukey’s test at the 5% significance level. Box plots display the median (line), mean (star), interquartile range (box), whiskers extending to 1.5 × IQR, and outliers (“+”).IQR: interquartile range. Treatment labels 0, 33, 66, and 99 correspond to 0, 0.33, 0.66, and 0.99% BME, respectively

Microbial diversity and taxonomic composition results are presented in Figs. 2, 3, 4 and 5. Barley malt extract supplementation did not affect overall fecal microbial alpha diversity, as indicated by the Chao1, corrected Chao1, Simpson, and Shannon indices (*P* > 0.05; Fig. 2).

Differences were observed in the relative abundance of specific taxa. The family Veillonellaceae was more abundant in piglets receiving 0.66% BME compared with those supplemented at 0.33% and 0.99% (*P* < 0.001; Fig. 3). Similarly, the genus *Mitsuokella* showed greater abundance at 0.66% inclusion, without differing from 0.99% (*P* = 0.034; Fig. 4). In contrast, the genus *Mogibacterium* was enriched at the highest supplementation level (0.99%) compared with the other treatments (*P* = 0.030; Fig. 5).

## Discussion

This study evaluated the effects of dietary inclusion of barley malt extract on growth performance, fecal consistency, immunological parameters, antioxidant status, short-chain fatty acid concentrations, and gut microbiota composition in nursery piglets. It was hypothesized that the high content of simple sugars and soluble proteins in BME would enhance nutrient availability and support post-weaning adaptation, thereby improving piglet post-weaning performance. Despite this rationale, no consistent dose–response effects were observed for growth performance variables. Body weight, average daily gain, feed intake, and feed conversion ratio were largely unaffected by increasing BME inclusion throughout the nursery period, except for a transient linear increase in FCR during the pre-starter II phase (8–28 days). These findings suggest that BME supplementation at the inclusion levels evaluated was insufficient to consistently modulate physiological adaptation or growth responses in nursery piglets.

These findings are consistent with reports of variable responses to barley-derived ingredients in pig diets. While malted barley grain has shown beneficial effects on nutrient digestibility and growth performance in growing–finishing pigs (Nasir et al. 2015), other barley-derived products, including brewery by-products and malt powders, have produced inconsistent or even negative effects on feed efficiency (Beyihayo et al., 2022). Similarly, Jin et al. (2019) reported reduced growth performance when barley replaced milk by-products in nursery pig diets, although reductions in diarrhea incidence were also observed. Such responses have been attributed primarily to soluble β-glucans and other structural carbohydrates capable of altering digesta viscosity, intestinal transit and microbial fermentation dynamics (Zheng et al. 2021). Likewise, Jha et al. (2010) demonstrated that barley cultivars differing in carbohydrate composition altered ileal digestibility and fermentation metabolites in weaned piglets, reinforcing the importance of substrate fermentability in shaping post-weaning intestinal responses (Su et al. 2026). In contrast, the BME used in the present study contained minimal fiber and β-glucans and consisted predominantly of simple sugars and soluble proteins. Therefore, the absence of performance responses observed here may be associated with compositional differences among barley-derived products, particularly regarding fiber content, carbohydrate structure, and fermentability. This distinction may be particularly relevant in nursery piglets, as gastrointestinal adaptation after weaning depends heavily on dietary substrates capable of supporting intestinal maturation, microbial stabilization, and fermentation processes (Han et al. 2024).

In addition to performance parameters, BME supplementation had minimal effects on diarrhea incidence, SCFA concentrations, and antioxidant status. Because simple carbohydrates are absorbed primarily in the proximal small intestine, rapidly digestible substrates such as those present in BME may provide limited fermentable material for microbial activity in the hindgut (Zhou et al. 2021; Zheng et al. 2021). Consequently, the absence of changes in SCFA concentrations and diarrhea incidence observed in the present study was biologically plausible, as fermentation-derived metabolites are highly dependent on the presence of complex fermentable substrates reaching the large intestine (Vasquez et al. 2022). Unlike whole or malted barley grains rich in non-starch polysaccharides (Martins et al. 2010; Zhong et al. 2015a), the BME used in the present study provided limited fermentable fiber. Supporting this interpretation, whole-grain barley has previously been shown to increase cecal SCFA production and modulate microbiota composition in animal models (Zhong et al. 2015b), emphasizing that the structural form and composition of barley-derived ingredients are likely important determinants of intestinal fermentation outcomes. Therefore, although BME supplementation did not compromise intestinal stability, combining BME with fermentable fiber sources could be evaluated in future studies to investigate possible synergistic functional effects.

Similarly, antioxidant enzyme activities and lipid peroxidation markers were unaffected by BME supplementation. These findings suggest that phenolic compounds present in BME were either insufficient in concentration or not sufficiently bioavailable to induce measurable systemic antioxidant effects. Although barley contains polyphenols with recognized antioxidant potential (Vitaglione et al. 2015), malting and extraction processes may alter the concentration, stability, or biological activity of these compounds (Koren et al. 2019). Therefore, any potential antioxidant effects of BME may have been limited by both ingredient composition and the relatively low inclusion levels evaluated in the present study.

Regarding immunological parameters, no treatment effects were observed for leukocyte populations. Period effects for neutrophils, lymphocytes, and basophils likely reflected expected physiological maturation during the post-weaning period rather than treatment-related responses. The absence of immunological differences among treatments suggests that BME supplementation neither stimulated nor impaired systemic immune function under the conditions evaluated. Importantly, leukocyte counts remained within physiological ranges reported for healthy swine (MSD Veterinary Manual, 2024), indicating no evidence of inflammatory challenge or immune suppression associated with dietary BME inclusion. Under the relatively stable sanitary and physiological conditions of the present study, potential immunomodulatory effects may have been more difficult to detect.

Although overall alpha diversity was not altered by BME supplementation, specific microbial taxa showed differential abundance. Veillonellaceae and *Mitsuokella* were enriched at intermediate inclusion levels, whereas *Mogibacterium* was more abundant at the highest level. Members of Veillonellaceae and *Mitsuokella* are commonly associated with carbohydrate metabolism and lactate utilization, suggesting that modest shifts in soluble substrate availability may have influenced microbial ecology (Jha and Berrocoso 2015; Zhou et al. 2021). However, these taxonomic changes were not accompanied by changes in SCFA concentrations, diarrhea incidence, or growth performance, indicating limited measurable functional consequences under the conditions evaluated. This finding highlights that alterations in microbial composition do not necessarily translate into detectable physiological or productive responses, particularly under relatively stable production and health conditions (Upadhaya and Kim 2022). Further studies integrating metabolomic approaches and host–microbiota interaction analyses may help clarify the biological relevance of these microbial shifts. From a practical perspective, the absence of detrimental effects suggests that dietary BME inclusion up to 0.99% is safe for nursery piglets, although measurable functional benefits were not observed under the conditions evaluated.

Some limitations should be considered when interpreting these findings. First, microbiota composition and SCFA concentrations were evaluated at a single timepoint, which may not fully reflect dynamic intestinal changes occurring throughout the nursery phase. Second, piglets were raised under controlled experimental nursery conditions without a specific sanitary or inflammatory challenge, potentially limiting the opportunity to detect functional responses to BME supplementation. Finally, the inclusion levels evaluated were selected to reflect commercially feasible dietary incorporation, and different supplementation strategies or combinations with fermentable ingredients may produce distinct outcomes. Future studies incorporating longitudinal sampling and conducted under commercial production conditions or defined health and environmental challenges may help clarify whether responses to BME vary throughout the nursery phase and across production contexts.

## Conclusion

Dietary inclusion of BME up to 0.99% did not improve growth performance, antioxidant status, immune parameters, intestinal fermentation, or overall microbial diversity in nursery piglets. Although a transient feed conversion response and modest alterations in specific microbial taxa were observed, these changes were not associated with measurable physiological or productive benefits. Under the conditions of this study, BME appeared to be functionally neutral as a standalone dietary ingredient during the nursery phase, neither impairing nor improving piglet performance. These findings contribute to the limited literature regarding barley malt-derived ingredients in swine nutrition by demonstrating that BME supplementation up to 0.99% should not be expected to improve growth performance or the evaluated physiological and intestinal outcomes under comparable nursery conditions. This evidence provides a practical benchmark for diet formulation and helps direct future research toward alternative supplementation strategies. Future research should evaluate alternative inclusion strategies or combinations with fermentable fiber sources to better define the functional role of BME in nursery pig nutrition.

## Supplementary Information

Below is the link to the electronic supplementary material.

Supplementary Material 1

Supplementary Material 2

## Data Availability

The data presented in this study are openly available in the University of São Paulo repository for dissertations and theses (*Teses USP*), at: https://doi.org/10.11606/T.10.2021.tde-07022022-103248

## Declarations

### Animal welfare

The authors confirm adherence to the journal’s ethical guidelines and that appropriate ethical review committee approval was obtained. The study was conducted in accordance with the VICH GL9 Good Clinical Practice guidelines for the protection of animals used in scientific research.
