# Supplementary material for: Effects of dietary barley malt extract on growth performance, immune parameters, intestinal fermentation and gut microbiota in nursery piglets
Source: Trop Anim Health Prod. 2026 Oct 10;58(8):587. doi: 10.1007/s11250-026-05388-w (PMC13653829; doi:10.1007/s11250-026-05388-w)
Supplement: Supplementary file 1 [file 11250_2026_5388_MOESM1_ESM.docx]

**SUPPLEMENTARY MATERIAL**

**Figure S1.** Average values of ambient temperature and relative humidity of the microclimate of the facility for piglets during the nursery period.
