# Supplementary material for: Effects of dietary barley malt extract on growth performance, immune parameters, intestinal fermentation and gut microbiota in nursery piglets
Source: Trop Anim Health Prod. 2026 Oct 10;58(8):587. doi: 10.1007/s11250-026-05388-w (PMC13653829; doi:10.1007/s11250-026-05388-w)
Supplement: Supplementary file 2 [file 11250_2026_5388_MOESM2_ESM.docx]

**HIGHLIGHTS**

- Graded inclusion of barley malt extract was evaluated in nursery piglets.
- BME up to 0.99% did not improve growth performance or feed efficiency.
- Immune parameters, antioxidant status and SCFA were unaffected by BME.
- Specific microbial taxa shifted without functional performance effects.
- Barley malt extract appears nutritionally neutral under nursery conditions.
